# Data showing the optimal conditions of pre-extraction and extraction of *Citrullus lanatus* (watermelon) white rind to increase the amount of bioactive compounds, DPPH radical scavenging and anti-tyrosinase activity

**DOI:** 10.1016/j.dib.2018.09.024

**Published:** 2018-09-12

**Authors:** Maryam Baeeri, Parisa Sarkhail, Ghazal Hashemi, Rezvan Marefatoddin, Zohre Shahabi

**Affiliations:** a Pharmaceutical Sciences Research Center (PSRC), The Institute of Pharmaceutical Sciences (TIPS), Tehran University of Medical Sciences, Tehran, Iran; b Students׳ Scientific Research Center, Faculty of Pharmacy, Tehran University of Medical Sciences, Tehran, Iran

## Abstract

In this data article, we examined some of those factors such as the effect of fresh, frozen and hot air-dried sample, pH and polarity of solvent by ultrasound-assisted extraction, as a “Green Extraction” technique, to find optimal conditions for increasing the amount of total phenolic and amino acid contents from watermelon rind. Then, we considered the DPPH radical scavenging and anti-tyrosinase activity of the extracts and their association with the amount of the phenolic and amino acid contents in the samples. The obtained data were analyzed one-way ANOVA, Tukey post hoc test and Graph Pad Prism 6 (*P* < 0.05). Our findings revealed one of the appropriate pre-extraction and extraction conditions of watermelon white rind to achieve more antioxidant and anti-tyrosinase effects. In addition, our data show the value of watermelon white rind as inexpensive, safe whitening and anti-browning agent, which can be used in pharmaceuticals, cosmetics and food products.

**Specifications table**

**Table:** 

Subject area	Biology and chemistry
More specific subject area	Byproduct (waste) development
Type of data	Table
How data was acquired	Ultrasonic bath, UV–vis microplate reader (Synergy HT, BIO-TEK, USA)
Data format	Raw data statistically analyzed
Experimental factors	Temperature, pH and polarity of solvents.
Experimental features	The watermelon white rinds were cut into small pieces and the same weighed kept in three different modes (pre-extraction technique).The samples extracted through three different solvents using an ultrasonic bath. Then the total phenolic content, amino acid content, DPPH free radical scavenging and mushroom-tyrosinase inhibition activity of the samples were evaluated.
Data source location	Pharmaceutical Sciences Research Center/Tehran University of Medical sciences, Tehran. Iran
Data accessibility	Data is with this article

**Value of the data**

• The data can be useful in providing the optimum pre-extraction and extraction conditions for increasing the stability and the level of phenolics and amino acid content in watermelon rind.
• The importance of pre-extraction technique in increasing the antioxidant activity of the watermelon white rind is described.
• Method and data presented in this article provide some information for increasing the anti-tyrosinase activity of the watermelon white rind.
• Our findings would be beneficial to the related researcher, on food additives, cosmetic and pharmaceutical production using watermelon white rind as a waste source.

## 1 Data

The data presented in this article show that the different temperatures in pre-extraction mode and solvent type (pH and polarity) can be influenced on the level of phenolic content, amino acid content, radical scavenging and anti-tyrosinase activity of watermelon white rind (Table 1). We considered the influence of the three unlike pre-extraction modes including, fresh mode, frozen mode and hot air-drying mode in early preparation of rind. In addition, in the extraction of samples, three different types of solvent were used with ultrasonic bath for 90 min.

**Table 1:** Total phenolic content (TPC), Total amino acid content (TAC), antioxidant capacity (DPPH radical scavenging) and anti-tyrosinase activity of fresh, frozen and hot air-dried sample of watermelon rind extracts.

**Sample name**	**TPC (mg equivalent Gallic acid/100 g sample)**	**TAC (mg equivalent Alanine/100 g sample)**	**DPPH radical scavenging (SC**_**50**_**(mg/mL))**	**Tyrosinase inhibition (IC**_**50**_**(mg/mL))**
WF	204.7 ± 11.26	81.7± 7.65	>200	> 200
WMF	691.4 ± 5.013	179.2 ± 2.20	>200	> 200
WAMF	878.6 ± 15.38	263.0 ± 8.58	>200	116.80
WFz	372.9 ± 17.32	393.2 ± 6.87	>200	> 200
WMFz	635.7 ± 21.90	674.0 ± 14.07	>200	> 200
WAMFz	813.1 ± 21.32	908.5 ± 7.37	>200	127.91
WD	2032 ± 22.99	470.0 ± 4.77	70.31	> 200
WMD	2443 ± 231.4	683.7 ± 41.33	54.67	> 200
WAMD	4290 ± 170.5	891.0 ± 19.09	58.72	33.46

Results are expressed as mean ± SEM. W: water, F: fresh, M: methanol, A: acetic acid, Fz: frozen, and D: dried samples.

## 2 Experimental design, materials, and methods

After removing the red fleshy pulp and the green rind of watermelon, the watermelon white rinds were cut into small pieces (20 mm by 10 mm). The similar weight of sample (100 g) kept in three different modes including, fresh mode (27 °C), frozen mode (−20 °C) and hot air- drying mode (60 °C). Then fresh and frozen samples crushed with a blender, while dried sample was milled. All samples were extracted using three different solvents: 1- water (W) 100%, 2- methanol: water (WM) (70:30); 3- methanol: water: acetic acid (WAM) (70:29:1) by an ultrasonic bath during 90 min. After filtration, the solvents were removed through vacuum or frieze dryer. All extracts were kept at −20 °C prior to experimental. The total phenolic content, total amino acid content, antioxidant and anti-tyrosinase activity of all samples were performed respectively, following method:

1. Folin-Ciocalteu׳s phenol reagent was used according to the method as previously described [1]. Briefly, the samples solution was incubated at room temperature with Folin and sodium carbonate for 60 minutes. The absorbance was measured at 760 nm using the ELISA reader. The results were expressed as milligrams of Gallic acid (GA) equivalents per 100 g of the each sample (mg GA/100 g sample weight).
2. The quantitation of free amino acid content was determined using the Ninhydrin reagent [2]. Briefly, after adding Ninhydrin solution to the samples solution, the tubes placed in boiling water bath for 10 minutes. Afterward the test tubes were cooled and 95% ethanol was added to each them. The absorbance of each solution read at 570 nm. The results were expressed as milligrams of Alanine (Ala) equivalents per 100 g of the each sample (mg Ala/100 g sample weight).
3. DPPH (1, 1-diphenyl-2-picrylhydrazyl radical) radicals reagent was used for evaluated the power of the free radical scavenging capacity of samples [1]. Briefly, samples solution with different concentrations was mixed with methanol solution of DPPH (100 µM). After 30 min incubation, the absorbance of the samples measured at 517 nm.
4. Mushroom tyrosinase assay was used to determine the power of tyrosinse inhibition of samples [1]. Briefly, concentrations of the samples solution mixed with phosphate buffer (50 mM) and mushroom tyrosinase solution (125 units)/mL. After pre-incubated at room temperature for 10 min, L-tyrosine (2 mM) was added into the reaction mixture and it was then incubated for. The absorbance was measured at 475 nm after 30 min at room temperature.
