# Supplementary material for: Data showing the optimal conditions of pre-extraction and extraction of Citrullus lanatus (watermelon) white rind to increase the amount of bioactive compounds, DPPH radical scavenging and anti-tyrosinase activity
Source: Data Brief. 2018 Sep 12;20:1683–5. doi: 10.1016/j.dib.2018.09.024 (PMC6157608; doi:10.1016/j.dib.2018.09.024)
Supplement: Supplementary file 1 — Transparency document [file mmc1.docx]

**Dear Editor:**

I am enclosing herewith a manuscript entitled “**Data showing the optimal conditions of pre-extraction and extraction of *Citrullus lanatus* (watermelon) white rind to increase the amount of bioactive compounds, DPPH radical scavenging and anti-tyrosinase activity**” for publication in **Journal of Data in Brief** for possible evaluation. The Corresponding author of this manuscript is Parisa Sarkhail and contribution of the authors as mentioned below with their responsibility in the research.

Maryam Baeeri, Ghazal Hashemi, Rezvan Marefatoddin, Zohre Shahabi

- The manuscript includes 2 references, 0 figures, 1 table, and 3 pages.
- The contents of this manuscript are not now under consideration for publication elsewhere.
- There are no directly related manuscripts or abstracts, published or unpublished, by any authors of this paper.
- The authors declare that there are no conflicts of interest.
- The authors declare that all the co-authors have agreed for submission Journal of Data in Brief
- The authors declare that the MS is prepared strictly according to the Journal format as provided in the instruction to authors.
- The authors declare that this article does not contain any studies with human or animal subjects.

Best regards

Sincerely

Dr. Parisa Sarkhail

Assistant Prof. in Pharmaceutical Sciences Research Center.

Tehran University of Medical Sciences (TUMS). Tehran. Iran.
